# Modulating rheology and bioactivity in dermal fillers: the emerging role of platelet-rich plasma (PRP)

**DOI:** 10.3389/fmed.2025.1728754

**Published:** 2026-01-06

**Authors:** Eduardo Anitua, Roberto Tierno, Mohammad Hamdan Alkhraisat

**Affiliations:** 1 BTI Biotechnology Institute, Vitoria, Spain; 2 University Institute for Regenerative Medicine and Oral Implantology - UIRMI (UPV/EHU-Fundación Eduardo Anitua), Vitoria, Spain; 3 Department of Oral and Maxillofacial Surgery, Oral Medicine and Periodontology, Faculty of Dentistry, University of Jordan, Amman, Jordan

**Keywords:** biostimulation, dermal filler, dilutional rheomodulation, platelet-rich plasma, regenerative therapy

## Abstract

This review explores the concept of dilutional rheomodulation in dermal fillers with the addition of platelet-rich plasma (PRP) and its potential to improve both aesthetic and regenerative outcomes. PRP is a biological product rich in growth factors and cytokines derived from the patient’s own blood, which plays a significant role in tissue regeneration and healing. According to previous studies that utilized titrated aqueous solutions as solvents, it is hypothesized that incorporating PRP into different dermal filler formulations may be effective for modulating the rheological parameteres of dermal fillers while providing regenerative and immunomodulatory properties, potentially improving biocompatibility, injectability, distribution, and overall tissue integration as suggested by preliminary investigations. This combined approach may reduce severe adverse effects associated with filler injections while enhancing their biostimulatory effects. Moreover, PRP has been shown to stimulate collagen production and promote skin regeneration, which may extend the filler’s longevity and improve skin texture and elasticity. Although early studies suggest positive outcomes, further clinical trials are needed to determine optimal dilution ratios, establish best practices, and assess long-term safety and efficacy. This review highlights the promising potential of PRP-filler combinations in advancing aesthetic procedures through the integration of immediate volumization with regenerative skin enhancement.

## Introduction

During the last decades, aesthetic and regenerative medicine have shifted focus from conventional structural interventions toward strategies that enhance endogenous self-repairing mechanisms (1). In this context, platelet-rich plasma (PRP) has gained attention as an autologous biological product with regenerative potential (2). PRP provides growth factors and cytokines—including PDGF, TGF-β, VEGF, EGF, IGFs, and FGFs—crucial for tissue repair, angiogenesis, immunomodulation, and extracellular matrix remodeling (3). Its use in dermatology and aesthetic medicine has grown significantly, supported by a strong safety profile and evidence for effectiveness in skin rejuvenation, alopecia, inflammatory dermatoses, and wound healing (4).

Injectable dermal fillers have also become essential for addressing age-related volume loss, facial sculpting, and dermal support (5). Their applications extend beyond cosmetic volume restoration to scar revision (6), lipodystrophy correction in HIV (7), post-traumatic or post-surgical contour defects (8), and regenerative purposes when combined with bioactive compounds (9). Soft-tissue fillers vary in composition, rheology, residence time, and biological interactions (10). These factors determine mechanical behavior—including viscoelasticity and cohesivity—which influence tissue integration, resistance to deformation, and biomechanical response (11). Additionally, biological behavior includes interactions with the extracellular matrix and immune system, biocompatibility, potential inflammatory or granulomatous reactions, and capacity to stimulate fibroblasts, induce neocollagenesis, or remodel tissue (12).

The longevity of a dermal filler denotes the duration over which it maintains clinical efficacy before enzymatic degradation or resorption, largely dependent on molecular composition, cross-linking, and physicochemical stability (13). Injectable implants can be broadly categorized into temporary, semi-permanent, and permanent fillers (14). Temporary fillers, commonly based on hyaluronic acid (HA), carboxymethylcellulose (CMC), polyethylene oxide (PEO) or collagen (bovine, porcine, or human), are biodegradable and well-tolerated, offering reversible results with a favorable safety profile (15). Semi-permanent fillers such as calcium hydroxylapatite (CaHA), high-density or cross-linked HA, and polyhydroxybutyrate (PHB) provide longer-lasting outcomes by maintaining mechanical volume and, in some cases, stimulating neocollagenesis while gradually resorbing (16). Permanent fillers, including polymethyl methacrylate (PMMA), acrylic hydrogels, polyalkylimide–polyacrylamide hydrogels, polyvinyl microspheres in polyacrylamide, e-polytetrafluoroethylene, Gore-tex, and autologous fat offer sustained volume but carry higher risks of adverse reactions (17). Autologous dermal fillers, a subclass of regenerative treatments, use the patient’s own tissue or cells—fat, dermis, platelet-rich fibrin (PRF), PRP, plasma-derived gel, or combinations—for facial rejuvenation and volumization. They generally provide more natural outcomes (18–25), though volume loss and fat harvesting complexity remain challenges. Nevertheless, they are promising options for personalized, non-synthetic treatments with lower complication risk (26).

In this context, it is critical to distinguish dermal fillers from biostimulators based on primary function (27). This distinction relates to their mechanism of action and physical properties. Classical dermal fillers, such as HA, CaHA, PMMA, or PHB products, are typically viscoelastic gels designed for rapid volume replacement via structural support. Primary biostimulators—including Poly-L-lactic acid (PLLA), Poly-D-lactic acid (PDLLA), polycaprolactone (PCL), or hyperdilute CaHA—are usually administered as colloidal suspensions with minimal or transient filling capacity, whose action is independent of viscoelastic properties. Their main function is to trigger controlled cellular signaling, gradually inducing neocollagenesis and yielding long-term volume restoration through tissue regeneration (28). While many injectables exhibit both structural and bioactive effects, classification depends on the dominant mechanism (29). Most autologous materials—PRP, plasma gel, or fat/dermal grafts—display a dual role: providing immediate or semi-permanent volume and structural support, while promoting tissue regeneration through fibroblast activation, neocollagenesis, and extracellular matrix remodeling, combining volumizing and bio-stimulatory effects (21, 22, 30).

Dilution of dermal fillers is a strategic approach aimed at modulating their rheological and biostimulatory properties (31–33). The choice of diluent—ranging from sterile water, isotonic saline or lidocaine to autologous components such as platelet-rich plasma (PRP) or extracellular matrix-derived solutions—significantly affects the structural integrity and biological performance of the filler (31, 34, 35). The use of platelet-rich plasma (PRP) as a diluent for dermal fillers may confer additional advantages, including enhanced biocompatibility, improved tissue integration, and stimulation of regenerative processes through the localized delivery of autologous growth factors (31, 36). The aim of this review is to synthesize the current state of knowledge and recent advances in the field of dilution-based rheological and biological modulation of dermal fillers and biostimulants using platelet-rich plasma (PRP). This approach, which involves modifying the rheological and biological properties of injectable biomaterials through dilution with autologous PRP, has emerged as a promising strategy to support filler biocompatibility, influence tissue integration, and possibly reduce adverse inflammatory responses. These combined effects are hypothesized to not only improve the clinical efficacy and aesthetic outcomes of filler treatments but also extend their durability and regenerative potential within the treated tissues.

## Results

### Dilutional rheomodulation of dermal fillers

The rheology of PRP is central to understanding its clinical performance and interactions with dermal fillers. Basic rheological parameters provide insight into specific viscoelastic responses. The elastic or storage modulus (G′) represents the portion of the deformation energy that is stored and then recovered during each oscillatory cycle, reflecting the material’s stiffness and solid-like behavior or resistance to deformation, while the loss or viscous modulus (G”) characterizes the flow-like component or the mechanical energy dissipated when the material undergoes structural changes (37). As summarized by McCarthy et al. (33), the loss tangent (tanδ) indicates the relative contribution of viscous and elastic behavior in each case, correlating with spreadability and fluidity. The complex modulus (G*) integrates elastic and viscous components, serving as an overall measure of gel strength. In addition, complex viscosity governs flow resistance, reflecting the material’s response to deformation and providing insight into its behavior during injection. Similarly, yield stress represents the minimum stress necessary to initiate flow, which is relevant for handling and injectability of dermal fillers and biostimulants, whereas cohesivity reflects the internal cohesion of the gel, influencing its ability to maintain shape and integrate with surrounding tissue. Collectively, these parameters provide a mechanistic framework for evaluating handling, injectability, and in vivo performance of dermal fillers and biostimulants. In this scenario, PRP exhibits unique viscoelastic behavior that is highly dependent on activation status and preparation conditions. Non-activated PRP and PRP-derived supernatants behave largely as Newtonian fluids, with low viscosity and minimal viscoelastic properties, offering negligible mechanical support when used alone (38–40). However, activation with calcium, thrombin, or collagen, as well as temperature variations, promotes fibrin polymerization, increasing G′ and overall gel stability.

The type of PRP activator and its combinations significantly influence both gel viscoelasticity and formation kinetics (41). For example, thrombin results in the firmest gels, characterized by the highest G′ and almost instantaneous gelation (42, 43). On the other hand, Calcium-based activation produces firm gels with moderately high G′ and gelation over 15–30 min (44–46), whereas collagen type I generates softer, more flexible gels with low G′ and much slower or minimal clot formation (47), highlighting that both activator selection or the combination of different activators dictate mechanical behavior and factor release.

The viscoelastic behavior of activated PRP is also correlated with both platelet and activator concentrations, with increased activator generally producing a more solid-like network, where elastic behavior (G′) dominates over viscous response (G″) (44). Moreover, temperature-based activation of PRP induces the formation of a fibrin network with enhanced G′ and sustained growth factor release, while thermal oscillation can transform liquid PRP into a dense, highly viscous paste with improved structural stability, potentially improving mechanical retention and bioactivity (48, 49). In addition, plasma gels formed through thermal treatment and albumin denaturation exhibit significantly increased viscoelasticity, with a solid-like network (high G′) that provides structural support and serves as a reservoir for bioactive molecules (21, 50, 51). As a result, the rheological properties of PRP can be precisely tailored by adjusting or combining different preparation methods and preparations, reflecting the broad textural versatility achievable through procedural variations that modulate its viscoelastic characteristics (21).

In this sense, evidence from composite hydrogel systems, including CaHA-CMC, CaHA-HA, and HA-PRP, demonstrates that dilutions with viscoelastic components produce nonlinear rheological effects that differ significantly from saline dilution (32, 33, 52–57). Accordingly, activated PRP-containing formulations are expected to form stronger, more cohesive composite gels than saline, non-activated PRP or PRP supernatant-diluted gels, with customizable rheological properties that allow precise modulation of filler performance and bioactive component delivery.

The timing of PRP activation – prior or subsequent to mixing with the filler or biostimulant- also represents a critical determinant of the rheological behavior of PRP-filler composites. Results obtained using PRP in its non-combined form have shown that it behaves primarily as a low-viscosity quasi-Newtonian protein solution until the onset of fibrin polymerization (39, 40), allowing for a more homogeneous mixture, lower extrusion force, and minimal early changes in viscosity, with fibrin matrix formation occurring *in situ* after filler injection. In contrast, PRP activation induces time-dependent fibrin polymerization and clot formation, leading to a swift increase in viscosity and cohesivity, higher extrusion forces, potential clogging, and non-uniform particle-fibrin distribution (42–44). Therefore, activation timing must be carefully considered when designing PRP-based composite gels to balance injectability, homogeneity, and rheological properties. However, the effects of PRP activation must be systematically assessed in each experimental context, since its interactions with diverse biomaterials are modulated by multiple factors, including the biomaterial’s concentration, particle size, surface topography and chemical composition, the dilution factor applied, the intrinsic cellular and biochemical profile of each specific PRP formulation, and the activation strategy adopted.

The rheological characterization of diluted injectables is essential for understanding their mechanical behavior, optimizing their delivery, and ensuring reproducibility in clinical applications. Table 1 provides an overview of key physical and rheological properties of the most common individual and composite dermal fillers and biostimulators, including their dilutions, as documented in the literature. Variations in viscosity, shear-thinning behavior, and viscoelasticity directly influence injectability, distribution in tissue planes, and cellular viability (58, 59). Comprehensive rheological profiling supports the standardization of protocols and enhances the predictability of graft integration and regenerative outcomes. Dilutional rheomodulation constitutes an emerging strategy in the modulation of dermal filler behavior, enabling precise control over their rheological properties (32). By diluting filler materials with isotonic solutions or bioactive agents —ranging from sterile water, saline or lidocaine to autologous components such as platelet-rich plasma (PRP) or extracellular matrix-derived solutions— it is possible to modulate critical parameters. From a biophysical perspective, dilution —when the filler’s viscoelasticity exceeds that of the diluent— reduces the filler’s viscosity and elastic modulus (G′), increasing spreadability, improving flow through fine-gauge needles and allowing for more uniform distribution across tissue planes (33). For example, the addition of PRP to hyaluronic acid (HA) results in a reduction of viscoelastic shear moduli and an increase in the crossover point, primarily attributable to a dilutional effect (53). This approach is particularly advantageous in treatments requiring subtle volumization or superficial placement, where high-viscosity gels may cause irregularities, overcorrection or overfilling (60, 61). Nevertheless, because the rheological parameters of PRP preparations can be extensively customized through procedural variations—including activation method, timing, incubation temperature, or the combination of different formulations—PRP dilution may be used to increase the viscoelasticity of low- to medium-viscoelasticity dermal fillers and biostimulatory agents, including collagen, low-density non-crosslinked HA, PLLA, and PDLLA.

**TABLE 1:** Physical and rheological properties, including dilutions, of common individual and composite dermal fillers and biostimulatory agents at the low-frequency regime.

Material	Dilution	G′ (Pa)^1^	G″ (Pa)^2^	G* (Pa)^3^	tanδ^4^	η* (Pa⋅s)^5^	Cohesivity^6^	Yield stress (Pa)^7^	References
CaHA-CMC + saline^8^	Undiluted	962	422.9	1050.85	0.44	≈2,000^13^	High	48	(32, 33, 57)
CaHA-CMC + saline^8^	1:0.25	113.2	92.49	146.18	0.817	≈100^13^	Low	5.7	(32, 33)
CaHA-CMC + saline^8^	1:0.5	40.78	42.61	59.98	1.045	≈90^13^	Low	2	(32, 33)
CaHA-CMC + saline^8^	1:1	8.369	12.42	14.977	1.484	≈25^13^	Low	0.42	(32, 33)
CaHA-CMC + saline^8^	1:2	1.021	2.512	2.712	2.46	≈2^13^	Low	0.05	(32, 33)
CaHA-CMC + saline^8^	1:3	0.119	0.6164	0.628	5.18	≈1^13^	Low	0.006	(32, 33)
PLLA^8^	Undiluted	0.015	NR	NR	NR	NR	NR	0.00075	(32)
HA^9^	Undiluted	6.93–603.14	8.79–91.7	11.19–584.87	0.15–1.27	149.09–1629.9	Low to high	0.35–30	(55, 57, 170, 171)
HA + CaHA entrapped (10% m/v)^10^	Undiluted	≈200^13^	≈20^13^	≈201^14^	≈0.10^14^	NR	NR	10	(55)
HA + CaHA entrapped (10% m/v)^10^	20% (m/v) CaHA free	≈300^13^	≈40^13^	≈303^14^	≈0.13^14^	NR	NR	15	(55)
HA + CaHA entrapped (10% m/v)^10^	45% (m/v) CaHA free	≈900^13^	≈100^13^	≈906^14^	≈0.11^14^	NR	NR	45	(55)
HA^10^	10% (m/v) CaHA entrapped	≈200^13^	≈20^13^	≈201^14^	≈0.10^14^	NR	NR	5.5	(55)
HA^10^	30% (m/v) CaHA entrapped	≈600^13^	≈70^13^	≈604^14^	≈0.12^14^	NR	NR	30	(55)
HA^10^	30% (m/v) CaHA free	≈300^13^	≈40^13^	≈303^14^	≈0.13^14^	NR	NR	6	(55)
HA + CaHA-CMC^10^	1:1	260.58–703.22	173.52–296.92	313.08–763.35	0.42–0.67	19.16–29.38	Medium	5–35	(52)
HA + CaHA-CMC^10^	1:2	139.81–495.83	104.9–183.13	174.79–528.58	0.37–0.75	20.18–27.74	Medium	7–25	(52)
HA + CaHA-CMC^10^	1:3	103.08–424.12	90.91–146.76	131.75–448.8	0.35–0.8	19.37–27.75	Low–medium	5–21	(52)
HA + CaHA-CMC^10^	1:4	86.31–388.95	71.16–126.16	113.5–408.9	0.32–0.86	19.61–28.43	Low–medium	4–19	(52)
PMMA-Collagen^11^	Undiluted	2815.27	NR	NR	NR	656.41	NR	141	(159)
PRP non-activated	Undiluted	NR	NR	<0.1^13^	NR	<0.01^13^	Low	<0.005	(38–40)
PRP activated^12^	Undiluted	≈5–80^13^	≈1–5^13^	≈3–80^13,14^	≈0.1–40^14^	NR	Low	0.4–4	(43, 44)

^1^*G*^′^(Pa), storage modulus; NR, not reported.

^2^*G*^″^(Pa), loss modulus.

^3^G*(Pa), complex modulus.

^4^tanδ, loss tangent.

^5^Complex viscosity η*(Pa⋅s).

^6^Cohesivity was classified based on G* and drop-test volume: low (∼200–400 Pa, >50 μL/drop), medium (∼400–800 Pa, 21–50 μL/drop), high (>800 Pa, 5–20 μL/drop) (11, 57).

^7^Yield stress (σ_*y*_) was estimated from the measured storage modulus (G′) under the assumption of a representative critical strain (γ ^c^ = 0.05) corresponding to the limit of the linear viscoelastic region, as commonly applied in soft gels and dermal fillers (172, 173). The calculation follows the relation σ_*y*_ ≈ G′⋅γ ^c^, providing an approximate, literature-supported value of the stress at which the material begins to yield. This approach allows comparison across different fillers and biostimulaory composites, while noting that exact yield stress should ideally be determined experimentally via amplitude sweep or creep tests.

^8^Rheological measurements evaluated at 0.1 Hz with a steady shear deformation of 0.1% under ambient conditions (25 °C).

^9^Rheological measurements were conducted at 0.1 Hz using small-amplitude oscillatory shear within the Linear Viscoelastic Region (LVER) at 37 °C.

^10^Rheological measurements evaluated at 1 Hz with a steady shear deformation of 0.1% under ambient conditions (25 °C).

^11^Rheological measurements evaluated at 0.7 Hz within the Linear Viscoelastic Region (LVER) under ambient conditions (25 °C).

^12^Rheological measurements evaluated at 0.1 Hz at 20 °C–25 °C.

^13^Data estimated from visual inspection of *G*^′^, *G*^″^, and η* curves; values are approximate and intended for comparative purposes.

^14^G* and tanδ were calculated from the primary moduli using the constitutive relationships: G* (Pa) = √(G′^2^ + G″^2^); tan δ = G″/G.

Dilution also increases the total filler volume and may promote a more uniform distribution of bioactive components, while the effect on injection precision depends on the resulting rheological properties of the mixture (62). Despite the generally favorable safety profile of dermal fillers, with most adverse events being mild and transient, rare but serious side effects have been documented, including infection, granuloma and vascular complications (63–69). Dilution-induced alterations in the rheological properties of dermal fillers can have significant implications for their safety profile, leading to a significant decrease in the amount of obstructive particles per volume of intravascular injectate and a reduction of the product’s cohesivity, resilience and elastic modulus, which are potential contributors to different mechanisms involving intravascular occlusion and external compressive forces (33, 56). Although the frequency of such adverse events is low, comprehensive safety assessment requires a large dataset derived from multiple studies involving different dermal fillers, dilution protocols, and clinical applications. Nevertheless, preliminary evidence suggests that individuals treated with diluted or hyperdiluted formulations may experience a lower rate of severe adverse reactions (70–74).

**FIGURE 1:**
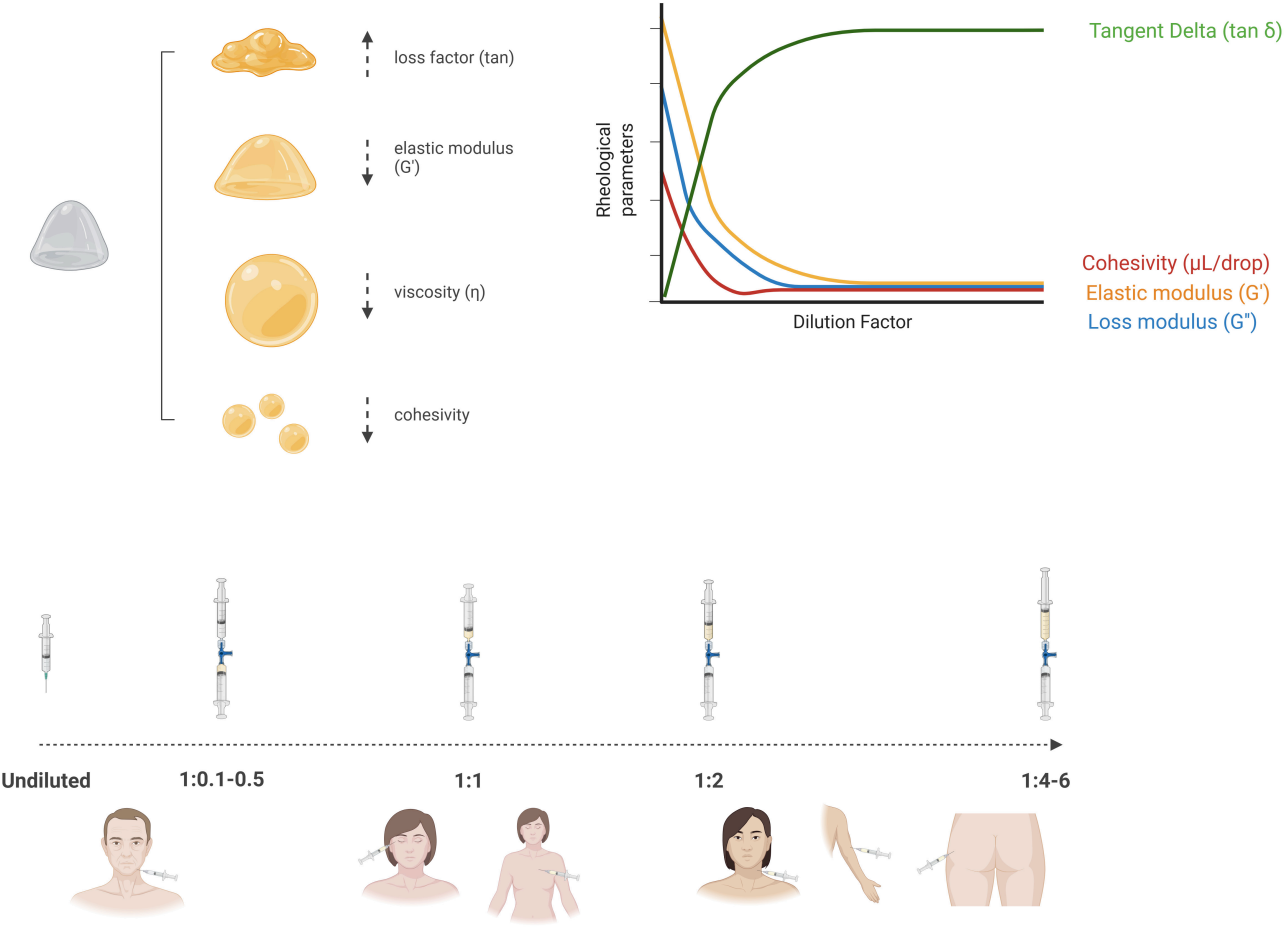
Schematic illustration of dilutional rheomodulation, depicting how controlled dilution modulates the viscosity and viscoelastic properties of a material to optimize its flow behavior and functional performance (Created in BioRender. Anitua, E. (2025) https://BioRender.com/11gkb15).

Figure 1 provides a synthesis of the concept of dilutional rheomodulation, illustrating its fundamental principles and highlighting its optimal applications in enhancing the rheological performance and clinical outcomes of dermal fillers. Most dilutional rheomodulation research has focused on investigating the effects of titrated aqueous solutions on rheological parameters of semi-permanent CaHA-based formulations combined with CMC (CaHA-CMC), a biphasic gel composed of suspended CaHA microspheres 25–45 μm in diameter (30% w/v) within a hydrophilic carrier (70% w/v), which includes water, glycerin and CMC (Radiesse, Merz Aesthetics, Raleigh, NC, United States) (70). In rheological terms, CaHA demonstrates higher G′ and viscosity values than other commercially available HA-based dermal fillers (75). Due to its high viscoelasticity, undiluted CaHA-CMC-based filler is optimally suited for deep dermal implantation and volumetric correction (57, 76, 77). Recommended treatment paradigm for diluted and hyperdiluted CaHA-CMC has been published by Goldie et al. (72), de Almeida et al. (78), Corduff et al. (79), Green et al. (80), ranging from undiluted CaHA-CMC (volume augmentation and structural support in areas like the chin, jawline, and temples) or diluted at 1:1 (facial rejuvenation, cellulite, striae or abdomen) to hyperdiluted 1:2–1:4 (laxity of the upper arm, neck or décolletage skin tightening or skin quality improvement) or even 1:6 as biostimulants (gluteal sagging or mild dermal irregularities on the buttocks).

According to Lorenc et al. (81), when CaHA-CMC is diluted at a 1:1 ratio with a biocompatible diluent, it retains partial volumizing effects due to the preservation of its intrinsic viscoelastic properties. However, at dilution ratios of 1:2 or higher, the CaHA-CMC biphasic filler undergoes a marked shift in rheological behavior, resulting in a substantial loss of volumizing capacity (32, 33, 77, 82). In this hyperdiluted form, CaHA primarily acts as a biostimulatory agent rather than a filler (72). The hyperdiluted formulation yields a suspension of CaHA microspheres that enables uniform distribution across broad anatomical regions (79). Rather than serving a volumizing function, hyperdiluted CaHA facilitates dermal regeneration by stimulating neocollagenesis and elastogenesis (83). Consequently, dilution protocols must be approached based on particle dispersion kinetics and cellular activation, rather than maintaining specific rheological properties required for structural fillers. This shift in functional behavior supports its role in regenerative aesthetic procedures, where the primary objective is to enhance skin quality—specifically elasticity, pliability, and dermal thickness—rather than to provide structural augmentation (73). Nevertheless, the dilution of dermal fillers remains an unstandardized practice, characterized by significant variability in dilution ratios, types of diluents, and clinical indications. This lack of consensus highlights the imperative for an evidence-based framework to guide dilutional protocols.

### Biological and clinical effects of dermal filler dilution

Despite the primary focus on traditional aqueous diluents such as sterile water, saline, lidocaine or lidocaine-epinephrine solutions, emerging attention is being directed toward novel formulations, such as poly-micronutrient-enriched solutions (84), exosomes (34), hyaluronidase or PRP (35). In this sense, non-activated PRP and PRP-derived supernatants demonstrate a basic rheological behavior similar to that of conventional aqueous diluents (38, 40). Its low viscosity and minimal elasticity facilitate the modulation of filler properties, enhancing injectability and distribution (39). Nevertheless, the rheological properties of PRP can be modulated through specific preparation parameters, including activation procedure, incubation time, temperature, and formulation strategy (21, 41–51). In addition, when PRP is utilized as a diluent for dermal fillers, the effects of dilutional rheomodulation can be synergistically enhanced through the inherent regenerative, anti-inflammatory and healing potential of the autologous platelet-rich derivative (35). Biostimulators can also be diluted to maximize the microparticle surface area available for cellular signaling, effectively shifting the primary mechanism from immediate structural support to widespread fibroblast stimulation and diffuse, long-term neocollagenesis (32, 33, 74, 85, 86). This dual functionality not only increases therapeutic efficacy but also reduces the risk of adverse effects related to immunogenicity (87) or contamination with impurities commonly associated with non-autologous fillers or diluents (88, 89). Therefore, PRP-based dilution of dermal fillers and biostimulants may help improve biocompatibility, potentially minimize adverse effects, and support tissue integration, ultimately contributing to optimized clinical outcomes in regenerative medicine applications.

### Hyaluronic acid-based fillers

Hyaluronic acid (HA) is a linear glycosaminoglycan naturally present in the extracellular matrix of vertebrate tissues, including connective, epithelial, and neural structures, with particularly high levels in the skin (90). Its capacity to bind water, along with its viscoelastic properties and biocompatibility with human tissue, has led to its widespread use as a dermal filler in aesthetic and reconstructive procedures (91). HA-based soft tissue fillers comprise several comercial formulations with particular characteristics (92), including Juvederm Ultra^®^, Juvederm Ultra Plus^®^ or Juvederm Voluma^®^ (Allergan Aesthetics an AbbVie Company, Irvine, CA, United States), Restylane^®^ Restylane Lyft^®^, and Restylane Silk^®^ (Galderma, Zug, Switzerland), Stylage^®^ (Laboratoires VIVACY, Archamps, France), Princess^®^ (Croma-Pharma GmbH, Leobendorf, Austria), and Belotero Balance^®^ (Merz Aesthetics, Raleigh, NC, United States) (93–95). When injected into the dermis or subcutaneous layers, HA helps restore soft tissue volume, reduce the appearance of wrinkles, and improve skin hydration (96). The results are temporary, as HA is progressively broken down by enzymes such as hyaluronidase, with clinical effects typically lasting up to 12 months, depending on the formulation and anatomical location (97, 98). Its favorable safety profile, reversibility, and low incidence of adverse immune responses have established HA as a cornerstone in minimally invasive facial rejuvenation (99).

Different combinations of PRP and HA-based injectable hydrogels have shown promising biological and clinical effects for facial rejuvenation (100) or skin defects management (101). A randomized controlled trial published by Hersant et al. (102) concluded that diluting HA-based filler (SkinVisc, Regen Lab, Switzerland) with autologous PRP (1:1) yielded enhanced outcomes in terms of skin elasticity and overall facial appearance when compared to the administration of either agent independently. Significant improvements in skin elasticity and skin smoothness have also been obtained using the combined PRP-HA technology for facial rejuvenation, which consists of tubes designed to prepare a mixture of PRP and non-crosslinked HA (103). Pirrello et al. (104) also demonstrated that filler injections of hyaluronic acid and PRP (A-CP HA kit, Regen Lab, Switzerland) in a similar proportion constitute an effective therapeutic option for patients with scleroderma considering both aesthetic appearance and functional improvement. Moreover, the hyperdilution of an HA filler (Tissuefill, JW Pharmaceutical, South Korea) with PRP (1:5) was reported to be effective and safe for facial augmentation (105). Analogous conclusions have been reached in studies aiming to evaluate the biological effect of HA-based hydrogels combined with PRP involving different therapeutic applications, including osteoarthritis management (106–108) or wound healing (109).

### CaHA-based fillers

CaHA is a naturally-occurring mineral that is commonly used as a biocompatible and biodegradable dermal filler with demonstrated efficacy in enhancing cutaneous structural integrity (69, 110). Its regenerative action includes activation of collagen and elastin synthesis, vascular neogenesis, and proliferation of dermal cells (78). Examples of commercially available CaHA-based dermal fillers include Hydroxyfill^®^ (Dr. Korman Laboratories Ltd., Kiryat Bialik, Israel), Radiesse^®^ (Merz Aesthetics, Raleigh, NC, United States) and HArmonyCa^®^ (Allergan Aesthetics an AbbVie Company, Irvine, CA, United States) (111). Although the potential synergy between CaHA and HA has been examined (112–114), the prevailing body of evidence centers around CaHA-CMC biphasic formulations. CaHA suspended in a CMC matrix demonstrates a strong regenerative potential over a wide dilutional range due to its capacity to stimulate resident fibroblasts (71, 85, 115, 116). The biophysical engagement between these fibroblasts and the CaHA microspheres plays a pivotal role in initiating new tissue synthesis (117). Notably, optimal regenerative effects have been associated with diluted preparations (from 1:1 to 1:3), likely attributable to the increased interparticle spacing, which facilitates cellular activity and matrix remodeling. This relationship has been confirmed through both laboratory experimentation and histological evaluations in clinical settings (118).

Clinical data largely support the effectiveness and safety profile of diluted CaHA-CMC at 1:3 or even lower dilutions for facial rejuvenation (119), soft tissue augmentation (120) or for the treatment of dorsal hand volume loss (121, 122), at 1:1 for cellulite dimpling on the buttocks (123), hyperdiluted CaHA-CMC (1:2) for the improvement of decollete wrinkles in females (124, 125), for the correction of volume loss in the infraorbital region (126), for skin rejuvenation (127) and also for skin tightening (128) and hyperdiluted CaHA-CMC (1:3) for the treatment of perioral rhytids (129). In the same line, improved neocollagenesis and neoelastogenesis have been reported after injection of diluted and hyperdiluted CaHA-CMC in different areas (71, 74, 122, 130, 131). Collagen neosynthesis has been consistently detected between 1 and 12 months following administration of CaHA-based fillers (71, 114, 132–134), including in treatments utilizing highly diluted filler solutions (74, 135).

Since standard aqueous solutions exhibit no inherent regenerative capacity, their replacement with bioactive solutions such as PRP has been proposed to enhance tissue regeneration and improve clinical outcomes. Khalifian et al. (35) concluded that a single session of a combination therapy based on hyperdiluted CaHA-CMC at a 1:4 dilution with a mixture of PRP and hyaluronidase was well-tolerated and demonstrated improvements in skin texture, along with a reduction in cervical rhytides and tissue laxity. According to the authors, the synergistic effect of PRP-derived growth factors and cytokines may enhance the regenerative potential of CaHA-CMC, facilitating fibroblast activation, collagen and elastin fiber biosynthesis, neo-angiogenesis and overall skin quality enhancement (136). Such biological mechanisms have been consistently demonstrated in clinical practice (137). Future investigations should further explore the role of PRP as a diluent in CaHA-CMC filler protocols, with systematic comparisons with conventional aqueous-based solutions.

### Other dermal fillers and biostimulants

Current literature provides limited insight into the biological and clinical consequences of diluting alternative injectable implant formulations. PLLA is an absorbable semi-permanent injectable biobased polymer that can be used to restore volume loss due to facial fat atrophy, while concurrently promoting dermal regeneration and skin texture improvement (138, 139). PLLA collagen-stimulators, including Sculptra^®^ (Galderma, Zug, Switzerland) and Lanluma V^®^ (Sinclair Pharma, London, United Kingdom), show a favorable safety profile, contribute to effectively increase volume, corrects laxity and improve contours, skin quality and the appearance of cellulite across various anatomical regions (140, 141). A trend toward higher reconstitution volumes in PLLA-containing injectable products has been observed in clinical practice, with lidocaine often added to the mixture to reduce injection-related discomfort (142–145). According to Palm et al. (146), the effectiveness of PLLA reconstituted with 8 mL of sterile water and 1 ml 2% lidocaine was similar to that of PLLA reconstituted with 5 ml of sterile water for the correction of nasolabial folds. To date, no published studies have been found addressing the use of PRP as a diluting agent for PLLA acid dermal fillers in the consulted literature. However, PRP-loaded PLLA-based biomaterials have demonstrated a strong regenerative potential in different soft tissue defect models (147, 148).

Collagen-based dermal fillers constitute a significant category of biodegradable non-permanent injectable materials in which the concept of dilutional rheomodulation, particularly involving PRP as an aqueous diluent or biostimulatory coadjuvant agent, remains under-investigated. Collagen is the predominant structural protein in vertebrate connective tissues, comprising approximately 30% of the total protein mass in the human body (149). The primary sources of collagen for the production of injectable formulations include bovine, porcine, swine, equine, and human tissues (150). Collagen-based dermal fillers, though available in numerous commercial forms with multiple origins and diverse biochemical profiles, present inherent challenges. These include susceptibility to enzymatic degradation, significant management and production costs, and risks associated with immunogenic responses and zoonotic disease transmission (151–155). To cite a few examples, Zyderm^®^ and Zyplast^®^ (Allergan, Dublin, Ireland), Cosmoplast^®^ and Cosmoderm^®^ (Inamed, Santa Barbara, CA, United States), GUNA^®^ (GUNA, Milan, Italy) products or CartiRegen^®^ (Joint Biomaterials, Mestre, Italy) (150). Regarding the effect of collagen dilution on dermal filler bioactivity, Weinkle (156), Yang et al. (157) demonstrated that the porcine-based dermal injectable collagen Dermicol-P35^®^ (Evolence, Ortho Dermatologics, Skillman, NJ, United States) combined with lidocaine constitute useful therapeutic strategies for correcting nasolabial fold wrinkles.

Polymethyl methacrylate is a non-biodegradable filler that, similar to CaHA and PLLA, exerts its effects primarily by stimulating de novo collagen synthesis (98, 158). PMMA-collagen gel is indicated for correcting moderate to severe acne scars, malar atrophy and infraorbital rhytides and for volume augmentation in regions such as the temple, chin, mandible, piriform aperture, nasolabial folds (98, 158–165). Although no studies have been identified that combine PMMA-based dermal fillers with PRP, experimental results confirm that the rheological properties of the carrier are critical for ensuring homogeneous distribution of the microspheres, which facilitates interstitial tissue infiltration (158, 166). Upon enzymatic degradation of the carrier, the microspheres persist in situ, functioning as a three-dimensional scaffold that supports neocollagenesis and tissue regeneration (58). In this sense, the rheological properties of collagen-PMMA gels limit their suitability for certain superficial applications in the undiluted form. As a result, the potential use of these gels as a diluted or hyper-diluted biostimulatory agent has been previously highlighted in the literature (159).

Although there is a gap in research regarding the use of PRP in collagen-based dermal filler formulations, several studies have shown that functionalizing collagen-based hydrogels or scaffolds with PRP improves cell proliferation, adhesion, migration, vascularization, and the deposition of mature collagen (167–169).

## Conclusion

This review consolidates current knowledge on PRP as a diluent for dermal fillers, examining theoretical rationale, clinical experience, and supporting evidence. It highlights limitations in existing data and suggests directions for further research in regenerative aesthetic medicine. Despite numerous studies addressing the role of dilutional rheomodulation in defining optimal therapeutic application of particular dermal fillers, they often limit analysis to clinical aptitude considering viscoelasticity, injectability, and filler volume, overlooking underlying biophysical factors. Future research should focus on elucidating effects of dilution on rheological parameters and therapeutic potential of dermal fillers from a biological perspective, with emphasis on autologous bioactive solutions such as PRP. Integration of PRP may enhance viscoelastic properties of the filler while minimizing immunogenic risks. Moreover, such combinations could provide the formulation with significant biostimulatory potential, promoting accelerated tissue regeneration, extracellular matrix synthesis, and neovascularization. Such integrative strategies may reinforce the physical resilience of dermal fillers and potentiate their role in tissue repair and remodeling.
